# Effect of Short-Chain Fatty Acids In Vivo on the Treatment of CCL_4_-Induced Hepatic Fibrosis

**DOI:** 10.3390/biomedicines14071477

**Published:** 2026-06-29

**Authors:** Kétlin Fernanda Rodrigues, Giovana Vivan Tonial, Matheus Scherer Bastos, Giovanna Mezzomo Pavanato, Carolina Luft, Maria Cláudia Rosa Garcia, Fábio Luiz Dal Moro Maito, Maria Martha Campos, Jarbas Rodrigues de Oliveira

**Affiliations:** School of Health and Life Sciences, Pontifical Catholic University of Rio Grande do Sul—PUCRS, Porto Alegre 90619-900, Brazil; ketlin.zrodrigues@gmail.com (K.F.R.); giovana.tonial@edu.pucrs.br (G.V.T.); matheus.bastos@acad.pucrs.br (M.S.B.); pavanatogiovanna@gmail.com (G.M.P.); carolina.luft@gmail.com (C.L.); maria.garcia@pucrs.br (M.C.R.G.); fmaito@pucrs.br (F.L.D.M.M.); maria.campos@pucrs.br (M.M.C.)

**Keywords:** hepatic fibrosis, short-chain fatty acids, hepatoprotection, inflammation

## Abstract

**Background/Objectives:** Hepatic fibrosis is a progressive pathological condition characterized by excessive extracellular matrix deposition in the liver, which may progress to cirrhosis and liver failure. Short-chain fatty acids (SCFAs) have demonstrated anti-inflammatory and anti-fibrotic properties; however, their therapeutic potential in hepatic fibrosis remains incompletely understood. This study aimed to evaluate the effects of acetate, propionate, and butyrate on carbon tetrachloride (CCl_4_)-induced hepatic fibrosis in Balb/C mice. **Methods:** Hepatic fibrosis was induced in Balb/C mice using CCl_4_, and the animals were treated with acetate, propionate, or butyrate administered via drinking water for four weeks. Biochemical parameters, histological alterations, and the expression of fibrogenic and inflammatory markers were evaluated and compared with a silymarin-treated group. **Results:** Treatment with SCFAs significantly reduced serum transaminase levels (AST and ALT) compared to the untreated fibrotic group. Histological analyses demonstrated the preservation of hepatic architecture and reduced inflammatory infiltrates, particularly in the butyrate-treated group. In addition, SCFAs significantly decreased the gene and protein expression of fibrogenic markers (ACTA2 and COL1A1) and inflammatory markers (NOS1, NF-κB, and IL-10), with propionate showing the most pronounced effects. Overall, the therapeutic effects observed with SCFAs were comparable or superior to those obtained with silymarin treatment. **Conclusions:** The findings suggest that SCFAs, especially butyrate and propionate, exert hepatoprotective, anti-inflammatory, and anti-fibrotic effects in CCl_4_-induced hepatic fibrosis. These compounds represent promising therapeutic candidates for the treatment of liver fibrosis.

## 1. Introduction

Hepatic fibrosis is a pathological condition characterized by the excessive accumulation of extracellular matrix components, particularly collagen, in the liver [1], resulting from chronic liver injuries. This process can progress to cirrhosis, a global health issue associated with severe complications such as portal hypertension and hepatocellular carcinoma [2]. Common etiologies include chronic viral hepatitis, alcohol abuse, and metabolic dysfunction-associated steatotic liver disease (MASLD) [3]. The progression of fibrosis involves complex interactions between hepatocytes, hepatic stellate cells, and inflammatory mediators, driven by oxidative stress and pro-inflammatory pathways [2].

Carbon tetrachloride (CCl_4_) is a well-established experimental model for inducing hepatic fibrosis in rodents, mimicking human chronic liver disease through the generation of reactive oxygen species [4]. Lipid peroxidation promoted by CCl_4_ leads to hepatocyte damage and fibrogenesis [5]. Balb/C mice are frequently used in these models due to their consistent fibrotic response, providing a reliable platform for evaluating therapeutic interventions [4].

Short-chain fatty acids (SCFAs), such as acetate, propionate, and butyrate, are microbial metabolites produced by the fermentation of dietary fibers in the gut [6]. These compounds exhibit anti-inflammatory, immunomodulatory, and antioxidant properties, which may attenuate liver injury and fibrosis [7]. Butyrate, in particular, reduces inflammation and oxidative stress in disease models by modulating histone deacetylase activity and G protein-coupled receptor signaling [8]. Evidence suggests that SCFAs influence hepatic homeostasis through the gut–liver axis, offering a promising therapeutic approach for hepatic fibrosis [6]. However, their specific effects on CCl_4_-induced hepatic fibrosis in Balb/C mice remain underexplored, warranting further investigation.

The objective of this study was to evaluate the therapeutic potential of short-chain fatty acids (acetate, propionate, and butyrate) in attenuating hepatic fibrosis induced by carbon tetrachloride in Balb/C mice, analyzing their effects on histological, biochemical, and molecular markers of hepatic fibrosis and inflammation.

## 2. Materials and Methods

The Male BALB/c mice, aged 60 days and weighing between 25 and 30 g, were used as the animal model. The animals were housed at the Center for Experimental Biological Models (CeMBE) of the Pontifical Catholic University of Rio Grande do Sul (PUCRS, Porto Alegre, Brazil) in ventilated cages under controlled conditions of temperature (22–24 °C) and a 12 h light/dark cycle. Throughout the experiment, the animals had ad libitum access to standard chow and water. The experimental design consisted of eight groups, each containing eight animals, defined as follows: Sham group (no fibrosis induction or treatment), Naive group (vehicle, receiving only olive oil as a solubilizer), CCl_4_ group (subjected to fibrosis induction with carbon tetrachloride), DMSO group (silymarin vehicle), CCl_4_ + DMSO group, CCl_4_ + silymarin group (200 mg/kg), and groups treated with short-chain fatty acids (acetate, propionate, and butyrate, each at 200 mM, administered via drinking water; Sigma-Aldrich, St. Louis, MO, USA).

To induce the hepatic fibrosis model, intraperitoneal administration of carbon tetrachloride (CCl_4_; Sigma-Aldrich, St. Louis, MO, USA) diluted to 20% in olive oil (Sigma-Aldrich) was performed at a dose of 1 mL/kg body weight, three times per week, for four consecutive weeks. Following the fibrosis induction period, the animals were treated with short-chain fatty acids (acetate, propionate, and butyrate) administered ad libitum via drinking water for an additional four weeks. At the end of the eight-week experimental protocol, the mice were euthanized with a lethal dose of a xylazine hydrochloride (Sigma-Aldrich) and ketamine solution (Sigma-Aldrich) (100 mg/kg) via intraperitoneal injection. Blood was collected by trunk puncture, and livers were excised for subsequent analyses. All animals received humane care, and the study protocols were conducted in accordance with institutional guidelines and applicable laws for animal research. The study was approved by the Institutional Animal Care and Use Committee of the University (protocol number 11474).

### 2.1. Assessment of Plasma Markers of Liver Injury and Function

Blood samples were collected and centrifuged at 4 °C and 5000 rpm for 10 min to obtain serum. Serum levels of aspartate aminotransferase (AST) and alanine aminotransferase (ALT), both markers of liver injury, were measured using a commercial kit from Labtest (Minas Gerais, Brazil) for the respective transaminase enzymes and quantified at 340 nm.

### 2.2. Histopathological Analysis

Liver tissue was fixed in 10% buffered formaldehyde solution and subsequently embedded in paraffin. Histological sections of 5 µm were obtained and subjected to different staining techniques. Hematoxylin and eosin (H&E) staining was used to assess the presence of inflammation. For detailed analysis of collagen fibers, sections were stained with Picrosirius Red (PSR). Slides were examined under optical microscopy by a pathologist blinded to the experimental design. Images were acquired using a BMX 43 microscope coupled to a DP73 digital camera (Olympus, Tokyo, Japan). Fibrosis severity was classified using semi-quantitative scoring systems, such as the METAVIR or Knodell scores, adapted for murine models. The presence of inflammatory infiltrates, hepatocellular necrosis, and alterations in lobular architecture was quantified in at least five microscopic fields per sample. Representative images were captured for documentation, and results were expressed as mean ± standard deviation. This analysis was based on adapted criteria [9]. The degree of inflammation was classified as follows: score 0 (no inflammatory foci), score 1 (one to two foci), score 2 (three to four foci), and score 3 (more than four foci). For fibrosis evaluation, classification was based on the location and extent of collagen deposition: score 1 (peri-sinusoidal fibrosis), score 2 (portal fibrosis), score 3 (combined peri-sinusoidal and portal fibrosis), score 4 (bridging fibrosis), and score 5 (established cirrhosis). This scoring system enabled a comparative assessment of the extent of hepatic fibrosis.

### 2.3. Gene Expression Analysis

mRNA expression was investigated using quantitative real-time PCR (qPCR) with the StepOne equipment (Applied Biosystems, Waltham, MA, USA) in liver tissue samples from animals subjected to the CCl_4_-induced fibrosis model. RNA was extracted using TRIzol™ reagent (Invitrogen, Carlsbad, CA, USA), followed by cDNA synthesis with the SuperScript III SuperMix kit (Invitrogen). RNA concentration was standardized to 5 µg across all samples. Relative expression of target genes was normalized to the reference gene beta-2-microglobulin (B2M). Reactions were performed using SYBR Green dye (Applied Biosystems). Data were expressed as ΔΔCT^2^, reflecting the variation in gene expression compared to the control group. To assess fibrosis-associated genes, primers for COL1A1 and ACTA2 were used, while primers for NOS1 and IL10 were employed for inflammatory markers. The sequences of the primers used in the gene expression analysis are listed in Table 1.

### 2.4. Protein Expression Analysis

Protein extraction was performed from mouse liver tissue using a lysis buffer composed of 0.5% CHAPS, β-mercaptoethanol, and protease inhibitors (Sigma-Aldrich, St. Louis, MO, USA). Samples were standardized to contain 40 µg of protein, separated by 10% (*w*/*v*) polyacrylamide gel electrophoresis, and transferred to a nitrocellulose membrane (Bio-Rad Laboratories, Hercules, CA, USA). The immunoblotting procedure included washes with PBS buffer containing 0.05% Tween (Sigma-Aldrich; Cat P9416) (PBST), followed by incubation in a blocking solution with 5% bovine serum albumin (BSA) in PBST for 30 min. Subsequently, the membrane was washed with PBST and incubated overnight at 4 °C in a blocking solution containing primary antibodies: anti-GAPDH (glyceraldehyde-3-phosphate dehydrogenase, 97166, Cell Signaling Technology, Danvers, MA, USA), anti-NF-κB p65 (nuclear factor kappa B, p65, #8242, Cell Signaling Technology), or anti-ACTA2 (alpha-smooth muscle actin, #19245, Cell Signaling Technology). After incubation, the membrane was washed and incubated for 2 h with a horseradish peroxidase (HRP)-conjugated secondary antibody. Subsequent washes with PBS were performed, and protein bands were visualized using a gel documentation system (Fujifilm, LAS-3000). The intensity of the bands was quantified using ImageJ software (version 1.54; National Institutes of Health, Bethesda, MD, USA).

### 2.5. Statistical Analysis

Data are presented as mean and standard deviation. One-way ANOVA was used, followed by Tukey’s post hoc test. *p*-values < 0.05, <0.01, and <0.001 are considered statistically significant. Statistical analyses were performed using GraphPad Prism 5.0 (GraphPad Software, San Diego, CA, USA). Data are expressed as mean ± SD (*n* = 6 minimum).

## 3. Results

### 3.1. SCFAs Demonstrate Hepatoprotective Effects by Reducing Transaminases in a Hepatic Fibrosis Model

**Figure 1:**
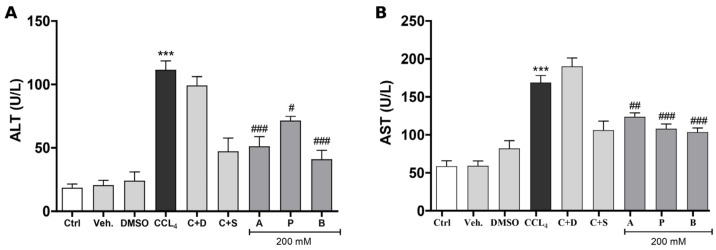
Effect of SCFAs on serological markers in CCl_4_-induced fibrotic mice. (**A**) ALT enzyme levels in mouse serum across treatment groups. (**B**) AST enzyme levels in mouse serum across treatment groups. Silymarin (200 mg/kg) was used as a positive control. Data represent the mean ± standard error of the mean (*n* = 8). *** *p* < 0.001 compared with the control group. # *p* < 0.05 compared with the CCl_4_ group. ## *p* < 0.01 compared with the CCl_4_ group. ### *p* < 0.001 compared with the CCl_4_ group. Control (control group); Vehicle (vehicle - olive oil); DMSO (dimethyl sulfoxide); CCl_4_ (carbon tetrachloride); C + D (CCl_4_ + DMSO); C + S (CCl_4_ + silymarin); A (CCl_4_ + acetate); P (CCl_4_ + propionate); B (CCl_4_ + butyrate).

Analyses of AST (aspartate aminotransferase) and ALT (alanine aminotransferase) showed that the induction of hepatic fibrosis by carbon tetrachloride (CCl_4_) (Figure 1A,B) significantly increased AST and ALT levels in the CCl_4_ and CCl_4_ + DMSO groups, reaching values of 125 ± 5 U/L and 210 ± 10 U/L, respectively, compared to the control group (25 ± 3 U/L for AST and 60 ± 4 U/L for ALT) and the vehicle group (50 ± 4 U/L for AST and 80 ± 5 U/L for ALT), indicating severe liver damage (*p* < 0.05). However, the groups treated with short-chain fatty acids exhibited a significant reduction in these markers. The CCl_4_ + silymarin group showed AST and ALT levels of 75 ± 6 U/L and 120 ± 8 U/L, respectively, while the groups treated with acetate, propionate, and butyrate displayed even lower values, with 50 ± 5 U/L and 80 ± 6 U/L. These data suggest that short-chain fatty acids have a significant hepatoprotective effect, attenuating CCl_4_-induced liver damage, with results comparable to or superior to silymarin, a well-known hepatoprotective agent.

### 3.2. SCFAs Attenuate CCl_4_-Induced Hepatic Fibrosis and Inflammation

**Figure 2:**
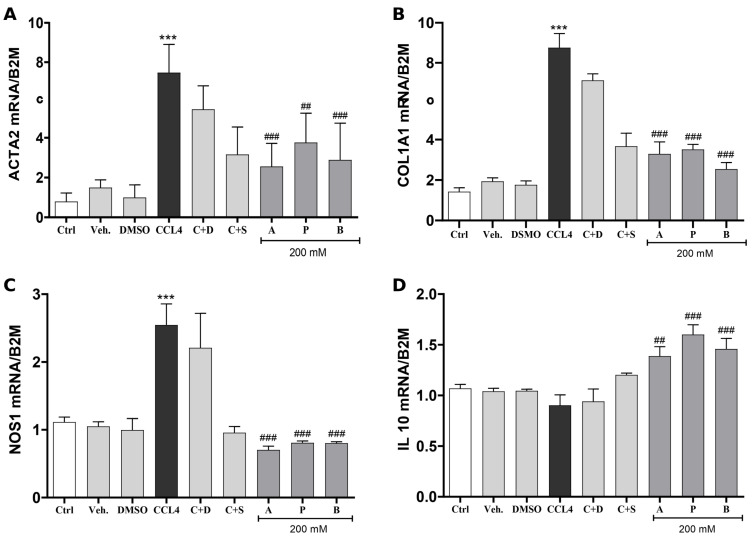
Effect of SCFAs on anti-fibrotic and anti-inflammatory markers in livers of CCl_4_-induced mice. mRNA expression of ACTA2 (**A**), COL1A1 (**B**), NOS1 (**C**), and IL10 (**D**) in treatment groups. Silymarin (200 mg/kg) was used as a positive control. B2M was used as an internal control for gene expression. Results are expressed as target gene/B2M, and data represent mean ± SD (*n* = 8) for gene expression. *** *p* < 0.001 compared with the control group, ## *p* <0.01 compared with the CCl_4_ group, ### *p* < 0.001 compared with the CCl_4_ group. Control (control group); Vehicle (vehicle—olive oil); DMSO (dimethyl sulfoxide); CCl_4_ (carbon tetrachloride); C + D (CCl_4_ + DMSO); C + S (CCl_4_ + silymarin); A (CCl_4_ + acetate); P (CCl_4_ + propionate); B (CCl_4_ + butyrate).

The induction of hepatic fibrosis by CCl_4_ resulted in a significant increase in the expression of fibrosis and inflammation markers, as assessed by RT-qPCR. The mRNA levels of ACTA2, COL1A1, NOS1, and IL10 were analyzed in the Sham, CCl_4_, Naïve, Silymarin, and groups treated with acetate (A), propionate (P), and butyrate (B) (200 mM). The CCl_4_ group exhibited a significant increase in ACTA2 expression compared to the Sham group. The Naïve and Silymarin groups significantly reduced this expression compared to the CCl_4_ group. Similarly, treatments with compounds A, P, and B also attenuated ACTA2 expression indicating an anti-fibrotic effect comparable to that of silymarin (Figure 2A).

COL1A1 expression was significantly elevated in the CCl_4_ group compared to the Sham group. Both the Naïve and Silymarin groups, as well as those treated with A, P, and B (200 mM), showed a significant reduction in COL1A1, suggesting decreased collagen deposition and fibrosis progression (Figure 2B). Likewise, NOS1 expression was significantly increased in the CCl_4_ group. All treated groups (Naïve, Silymarin, A, P, and B) significantly reduced NOS1 levels, demonstrating a robust anti-inflammatory effect (Figure 2C). Regarding IL10 expression, the CCl_4_ group exhibited significantly increased levels compared to the Sham group, possibly reflecting a compensatory anti-inflammatory response to hepatic injury and inflammation. Treatment with silymarin and SCFAs reduced IL10 expression compared to the CCl_4_ group, particularly in the propionate- and butyrate-treated groups. This reduction may be associated with the attenuation of the inflammatory process and decreased requirement for compensatory IL10 upregulation (Figure 2D).

Overall, compounds A, P, and B demonstrated anti-fibrotic and anti-inflammatory effects similar to those observed in the Silymarin-treated group, significantly attenuating the expression of ACTA2, COL1A1, NOS1, and IL10 genes. No statistical differences were observed among compounds A, P, and B, indicating that all exhibit comparable therapeutic potential in the CCl_4_-induced hepatic fibrosis model.

### 3.3. SCFAs Attenuate Markers of CCl_4_-Induced Hepatic Fibrosis and Inflammation

**Figure 3:**
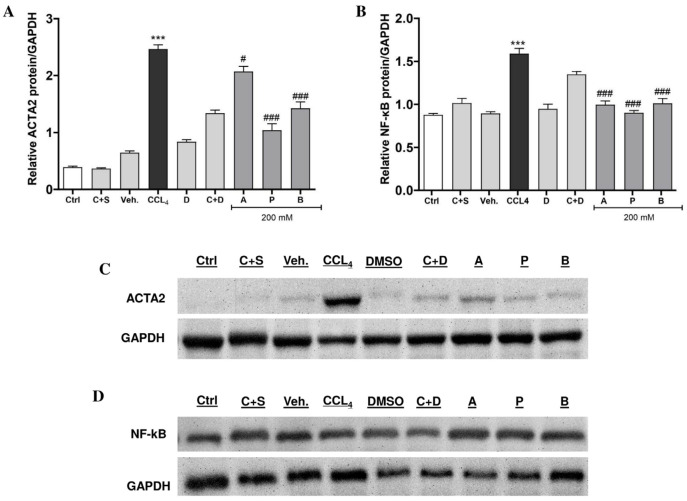
Effect of SCFAs on anti-fibrotic and anti-inflammatory markers in livers of CCl_4_-induced mice evaluated by Western blot protein expression. Protein expression of ACTA2 (**A**,**C**) and NF-κB (**B**,**D**) in treatment groups. Silymarin (200 mg/kg) was used as a positive control. GAPDH was used as an internal control for protein expression. Results are expressed as target protein/GAPDH, and data represent mean ± SD (*n* = 8) for protein expression. *** *p* < 0.001 compared with the control group, # *p* < 0.05 compared with the CCl_4_ group, ### *p* < 0.001 compared with the CCl_4_ group. Control (control group); Vehicle (vehicle—olive oil); DMSO (dimethyl sulfoxide); CCl_4_ (carbon tetrachloride); C + D (CCl_4_ + DMSO); C + S (CCl_4_ + silymarin); A (CCl_4_ + acetate); P (CCl_4_ + propionate); B (CCl_4_ + butyrate).

To evaluate the effect of short-chain fatty acids on the treatment of hepatic fibrosis, we analyzed the expression of ACTA2 protein by Western blot, using GAPDH as a loading control. Animals treated with CCl_4_ exhibited a significant increase in ACTA2 expression compared to the control group, indicating the development of fibrosis. The CCl_4_ + DMSO group also maintained elevated ACTA2 levels, demonstrating that the vehicle did not interfere with the fibrotic process (Figure 3A,C). Administration of silymarin resulted in a significant reduction in ACTA2 expression compared to the CCl_4_ group, confirming its well-established hepatoprotective effect. Similarly, treatment with short-chain fatty acids (SCFAs)—acetate, propionate, and butyrate—also significantly reduced ACTA2 expression compared to the CCl_4_ group, suggesting an anti-fibrotic effect of these compounds. Among the evaluated SCFAs, propionate demonstrated the highest efficacy in inhibiting ACTA2 expression, followed by butyrate and acetate. These results indicate that SCFAs, particularly propionate, may exert therapeutic effects in CCl_4_-induced hepatic fibrosis models (Figure 3A,C).

The expression of NF-κB protein was assessed by Western blot as a marker of the inflammatory response associated with hepatic fibrosis. Animals treated with CCl_4_ showed a significant increase in NF-κB expression compared to the control group, indicating the activation of the inflammatory pathway. The CCl_4_ + DMSO group exhibited a similar pattern, suggesting that the vehicle does not interfere with NF-κB activation (Figure 3B,D).

Treatment with silymarin resulted in a significant reduction in NF-κB expression compared to the CCl_4_ group. Similarly, the short-chain fatty acids—acetate, propionate, and butyrate—also significantly reduced NF-κB expression, suggesting an anti-inflammatory effect of these compounds. These results indicate that SCFAs, like silymarin, are capable of attenuating the activation of NF-κB-mediated inflammatory pathways in an experimental model of CCl_4_-induced hepatic fibrosis (Figure 3B,D).

### 3.4. SCFAs Attenuate CCl_4_-Induced Hepatic Histological Lesions

**Figure 4:**
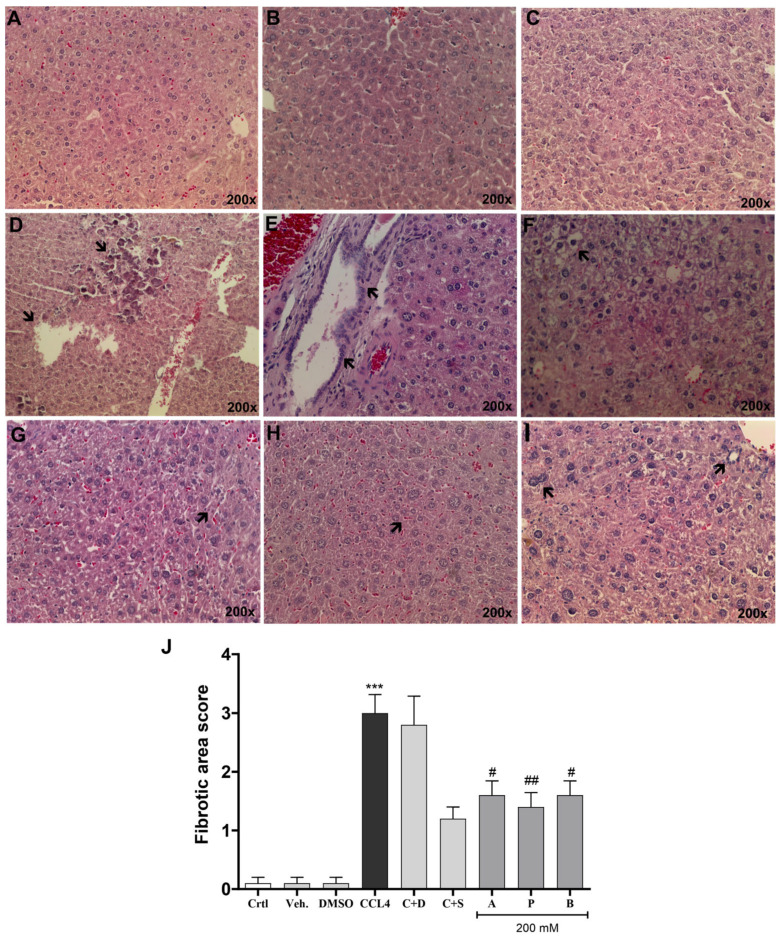
Histopathological assessment of hepatic injury across experimental groups. Effects of SCFAs on hepatic inflammation. Lobular inflammation was evaluated by H&E staining in the control group (**A**); vehicle group (**B**); DMSO group (**C**); CCl_4_ group (**D**); CCl_4_ + DMSO (C + D) group (**E**); CCl_4_ + 200 mg/kg silymarin (C + S) group (**F**); CCl_4_ + acetate (A) group (**G**); CCl_4_ + propionate (P) group (**H**); CCl_4_ + butyrate (B) group (**I**) Assessment of hepatic inflammatory infiltrate (**J**). Black arrows indicate areas of inflammatory cell infiltration. Magnification 200×. Quantification of the lobular inflammation score. Data represent the mean ± SD (*n* = 8). *** *p* < 0.001 compared with the control group, # *p* < 0.05 compared with the CCl_4_ group, ## *p* < 0.01 compared with the CCl_4_ group.

Histological analysis of livers stained with hematoxylin and eosin (H&E) revealed significant morphological changes among the experimental groups. The control groups (Sham and Naïve) exhibited preserved hepatic architecture, with no signs of degeneration, inflammation, or fibrosis (Figure 4A,B). The silymarin-treated group (Figure 4C) also showed preserved hepatic parenchyma with mild congestion, indicating a hepatoprotective effect. In contrast, the CCl_4_-induced groups (Figure 4D,E) displayed extensive areas of necrosis, dense inflammatory infiltrates, loss of lobular architecture, and vascular congestion. The groups treated with SCFAs (acetate, propionate, and butyrate—Figure 4F–I, respectively) exhibited varying degrees of histological improvement. The butyrate-treated group (Figure 4H) demonstrated preserved architectural structure and minimal inflammatory infiltrates, while the propionate (Figure 4H) and acetate (Figure 4G) groups showed moderate improvement with reduced necrosis and inflammation.

**Figure 5:**
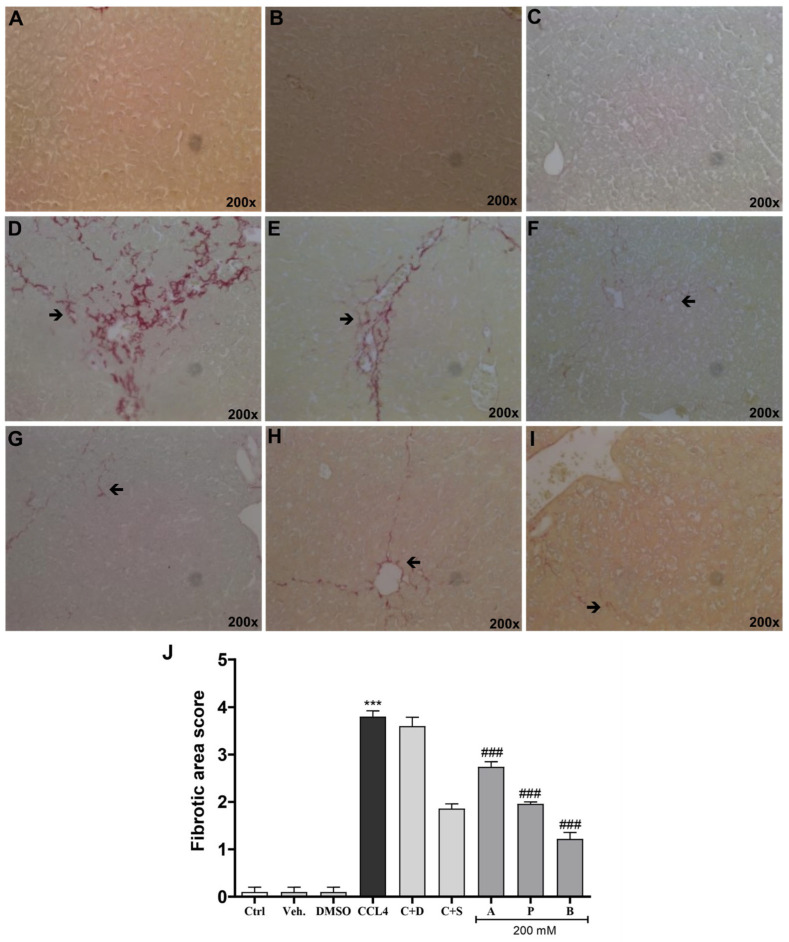
Histopathological assessment of hepatic injury across experimental groups. Effects of SCFAs on hepatic fibrosis. Collagen fibers were evaluated by Picrosirius Red staining in the control group (**A**); vehicle group (**B**); DMSO group (**C**); CCl_4_ group (**D**); CCl_4_ + DMSO (C + D) group (**E**); CCl_4_ + 200 mg/kg silymarin (C + S) group (**F**); CCl_4_ + acetate (A) group (**G**); CCl_4_ + propionate (P) group (**H**); CCl_4_ + butyrate (B) group (**I**) Quantification of hepatic collagen deposition (**J**). Black arrows indicate the presence of collagen fibers. Magnification 200×. Quantification of the collagen deposition score. Data represent the mean ± SD (*n* = 8). *** *p* < 0.001 compared with the control group, ### *p* < 0.001 compared with the CCl_4_ group.

Complementing these findings, Picrosirius Red staining revealed significant histological changes in the livers of mice from different experimental groups. The control group (Figure 5A) exhibited preserved hepatic architecture with no collagen deposition, similar to the vehicle (Figure 5B) and DMSO (Figure 5C) groups. In contrast, the CCl_4_ group (Figure 5D) displayed extensive hepatic fibrosis, evidenced by intense collagen deposition around centrilobular veins and portal spaces (red arrows), indicating the disruption of hepatic architecture. The CCl_4_ + DMSO group (Figure 5E) showed a similar fibrosis pattern, suggesting that DMSO had no protective effect against CCl_4_-induced fibrosis. Treatment with silymarin (Figure 5F) resulted in a notable reduction in collagen deposition, confirming its hepatoprotective effect. Among the SCFA-treated groups, the acetate, propionate, and butyrate groups (Figure 5G–I) exhibited a significant reduction in hepatic fibrosis compared to the CCl_4_ group, with butyrate showing the most pronounced preservation of hepatic architecture and reduction in collagen deposition. Quantification of hepatic lesions and collagen deposition was performed using a histopathological scoring system, evaluating parameters such as necrosis, inflammation, congestion, tissue disorganization, and collagen fiber presence on a scale from zero to four. The SCFA-treated groups showed lower scores compared to the CCl_4_ group, with the butyrate group achieving the best outcome. The Sham and Naïve groups had a score of zero. Silymarin slightly reduced the score. The CCl_4_ group exhibited the maximum score (four), indicating severe injury. Treatments with acetate, propionate, and butyrate significantly reduced the score, with butyrate being the most effective, approaching control levels. Total scores (necrosis, inflammation, congestion, and tissue disorganization; scale 0–4) were quantified by blinded analysis using Prism (mean ± SD, n = 8 samples per group).

## 4. Discussion

The results of this study demonstrate that short-chain fatty acids (SCFAs) at a concentration of 200 mM exhibit significant hepatoprotective, anti-fibrotic, and anti-inflammatory effects in a carbon tetrachloride (CCl_4_)-induced liver fibrosis model in male Balb/C mice. These effects were comparable, and in some cases superior, to those observed with silymarin, a widely used hepatoprotective agent [10]. The significant reduction in transaminase levels (AST and ALT), expression of fibrotic (ACTA2 and COL1A1) and inflammatory (NOS1 and IL 10) markers, as well as the observed histological improvement, corroborate the therapeutic potential of SCFAs [11].

The ACTA2 and COL1A1 genes are key biomarkers in the study of fibrosis and fibroblast phenotype. The simultaneous activation of these two cellular pathways is strongly associated with wound healing responses, tissue stiffness, and fibrotic pathologies. The decreased expression of ACTA2 and COL1A1 in SCFA-treated groups suggests the attenuation of hepatic stellate cell (HSC) activation, a central event in the progression of hepatic fibrosis [12]. HSC activation leads to transdifferentiation into myofibroblasts, which promote extracellular matrix deposition, particularly type I collagen [13]. Our findings indicate that SCFAs may interfere with this process, possibly through the inhibition of histone deacetylases (HDACs); butyrate has been shown to modulate the expression of fibrotic genes via HDAC inhibition in fibrosis models, which may explain the observed effects [14]. Additionally, the activation of G protein-coupled receptors, such as GPR41 and GPR43, may mediate the anti-fibrotic effects of SCFAs by regulating inflammation and hepatic homeostasis [15].

These molecular changes were accompanied by consistent histological findings. CCl_4_ administration induced severe liver injury, with extensive necrosis, intense inflammation, vascular congestion, loss of lobular architecture, and marked collagen deposition, as evidenced by hematoxylin and eosin (H&E) and Picrosirius Red staining [16]. Silymarin treatment provided partial hepatic protection, preserving tissue architecture and reducing collagen deposition, confirming its hepatoprotective effect [17]. Clinical evidence also suggests that silymarin can reduce liver stiffness and improve biochemical parameters in patients with hepatic steatosis, possibly via modulation of the gut microbiota [18].

Similarly, SCFAs promoted significant histological improvement, with reductions in necrosis, inflammation, and collagen deposition. Semi-quantitative analysis demonstrated that all SCFAs reduced histopathological scores compared to the CCl_4_ group, with butyrate being the most effective in reversing inflammatory and structural changes. These results align with the literature, which highlights butyrate as the SCFA with the greatest anti-inflammatory and antioxidant potential due to its affinity for GPCRs and its ability to influence hepatic homeostasis via the gut–liver axis [19]. The NOS1 and NF-κB genes promote inflammation, while IL-10 acts as an anti-inflammatory agent. NOS1 promotes the inflammatory activation of NF-κB, while IL-10 acts as an immune brake, suppressing this same pathway to protect tissues. The modulation of NF-κB, NOS1, and IL-10 markers further supports this role. The increase in NOS1 in the CCl_4_ group reflects oxidative stress and exacerbated inflammatory activation [20]. The significant reduction in NOS1 with SCFAs suggests the inhibition of the NF-κB pathway, a key regulator of inflammation associated with hepatic fibrosis [21]. Although IL-10 is classically recognized as an anti-inflammatory cytokine, its increased expression in the CCl_4_ group may reflect a compensatory response to intense hepatic inflammation and tissue injury. The reduction in IL-10 expression observed in SCFA-treated groups, particularly with propionate and butyrate, may therefore indicate the attenuation of the inflammatory environment rather than the suppression of anti-inflammatory signaling itself. This interpretation is supported by the concomitant reduction in NOS1 and NF-κB expression, as well as the histological improvement observed in treated animals. Previous studies have demonstrated that SCFAs are capable of modulating macrophage activation and inflammatory signaling pathways, contributing to the restoration of hepatic homeostasis [22,23].

Histopathological analysis confirmed that butyrate was the most effective SCFA in preserving hepatic architecture and reducing inflammatory infiltrates, followed by propionate and acetate. Interestingly, while butyrate showed overall superiority, propionate exhibited comparable performance in inhibiting ACTA2, suggesting that different SCFAs may act through distinct mechanisms [15]. Notably, DMSO, used as a vehicle, did not significantly alter histological parameters or collagen deposition, confirming its neutrality in the experimental model [24].

The clinical implications of these findings are promising. Supplementation with dietary fibers or SCFA-containing formulations may represent an adjunctive therapeutic strategy for chronic liver diseases, such as non-viral cirrhosis and hepatitis, characterized by persistent inflammation and tissue remodeling [25]. The administration of SCFAs via drinking water, as performed in this study, mimics the endogenous intestinal release of these metabolites, suggesting that prebiotic-rich diets may increase their circulating levels [19]. However, aspects such as bioavailability, optimal dosage, and tolerability in humans require further clinical investigation. This study has some limitations. Experiments were conducted exclusively in male Balb/C mice; therefore, the influence of sex-related factors, such as estrogen’s known hepatoprotective properties, on the observed effects of SCFAs remains to be investigated in female animals. Additionally, a single SCFA concentration was tested and the CCl_4_ model, while widely used, may not fully recapitulate human chronic liver disease.

In summary, the results of this study reinforce the therapeutic potential of SCFAs, particularly butyrate, as hepatoprotective and anti-fibrotic agents. Their ability to modulate epigenetic, inflammatory, and fibrotic pathways highlights them as promising candidates for nutraceutical and pharmacological approaches [11]. Future clinical studies will be essential to validate their efficacy, safety, and applicability in patients with chronic liver diseases.

## 5. Conclusions

This study demonstrates that short-chain fatty acids (acetate, propionate, and butyrate) exhibit significant hepatoprotective, anti-fibrotic, and anti-inflammatory effects in a CCl_4_-induced hepatic fibrosis model in male Balb/C mice. These compounds attenuated hepatic stellate cell activation, reduced collagen deposition, and modulated hepatic inflammation, with efficacy comparable to or superior to that of silymarin. Butyrate stood out as the most effective SCFA in preserving hepatic architecture, while propionate showed greater potency in inhibiting fibrotic markers such as ACTA2. These findings reinforce the therapeutic potential of SCFAs in the treatment of hepatic fibrosis, suggesting that strategies based on fiber-rich diets or SCFA supplementation may be promising for chronic liver diseases. Further studies are needed to validate these results in other experimental models and clinical trials, as well as to elucidate the underlying molecular mechanisms.

## Figures and Tables

**Table 1:** Primer sequences used for gene expression analysis. The table lists the gene name (column 1), the Forward (F) and Reverse (R) primer sequences (column 2).

Gene	Primers Sequences (5′-3′)
*B2M*	F: ACAGTTCCACCCGCCCTCACATT
	R: TAGAAAAGAGCACAGTTCCTTGCTGAAG
*ACTA2*	F: TAGCCACCGAGCACCATGAAG
	R: CTGCTGGAAAGGTGGACAGAG
*COL1A1*	F: AGTGGTTTGGATGGTTGCCA
	R: GCACCCATCATTTCCACGGAGC
*NOS1*	F: CCTCCTCAGCCCTACCCAAAGT
	R: CACCCAAAGTGCTTCAGTCA
*IL 10*	F: ATCCTGACTTCTTTTCCTTG
	R: GCCTTCTTTTGCAAGTCTGTC

B2M: Beta-2-microglobulin; ACTA2: Actin, alpha 2, smooth muscle; COL1A1: Collagen, type I, alpha 1 chain; NOS1: Nitric oxide synthase 1; IL10: Interleukin 10.

## Data Availability

The data presented in this study are available on request from the corresponding author. The data are not publicly available due to ongoing research and publication purposes.

## Abbreviations

SCFAs	Short-Chain Fatty Acids
CCl_4_	Carbon Tetrachloride
AST (TGO)	Aspartate Aminotransferase
ALT (TGP)	Alanine Aminotransferase
H&E	Hematoxylin and Eosin
PSR	Picrosirius Red
qPCR	Quantitative Polymerase Chain Reaction
RT-qPCR	Reverse Transcription Quantitative PCR
mRNA	Messenger Ribonucleic Acid
COL1A1	Collagen Type I
ACTA2	Alpha-Smooth Muscle Actin
NOS1	Inducible Nitric Oxide Synthase
IL 10	Interleukin 10
B2M	Beta-2-Microglobulin
GAPDH	Glyceraldehyde-3-Phosphate Dehydrogenase
NF-κB	Nuclear Factor kappa B
HRP	Horseradish Peroxidase
PBST	Phosphate-Buffered Saline with Tween
BSA	Bovine Serum Albumin
HDACs	Histone Deacetylases
GPR41/GPR43	G Protein-Coupled Receptor 41/43
NLRP3	NOD-Like Receptor Family, Pyrin Domain Containing 3
CeMBE	Center for Experimental Biological Models
PUCRS	Pontifical Catholic University of Rio Grande do Sul
SD	Standard Deviation
ANOVA	Analysis of Variance
SEM	Standard Error of the Mean
